# Improving Emergency Referrals in a Philippine Tertiary Hospital: A Quality Improvement Initiative

**DOI:** 10.7759/cureus.86394

**Published:** 2025-06-19

**Authors:** Eron Allen Tan

**Affiliations:** 1 Health Sciences Program, Ateneo de Manila University, Quezon City, PHL

**Keywords:** documentation compliance, emergency documentation compliance, emergency referral system, family medicine, outpatient clinic workflow, philippine outpatient emergency care, public hospital workflow, quality improvement, quality improvement in hospitals, referral turnaround time

## Abstract

Introduction

Emergency referrals from outpatient clinics to emergency departments (EDs) are critical in managing acute medical conditions. This quality improvement (QI) study at a tertiary public hospital in the Philippines addressed delays and documentation issues in the emergency referral process. This study aimed to improve referral turnaround time and compliance through a structured quality improvement initiative.

Methods

A retrospective before-and-after quality improvement study was conducted from March to June 2023. Twenty referrals before and 20 after interventions were audited. Interventions included protocol training, assignment of a triage resident, structured verbal endorsement, nursing coordination, and a standardized referral checklist. The outcomes measured were turnaround time and documentation compliance.

Results

The average referral time decreased from 43.05 minutes to 15.75 minutes post-intervention, a 63% improvement. Documentation compliance improved from 75.15% to 98%. All post-intervention cases had complete vital signs, medical history, physical examination notes, and formal endorsement. Staff reported greater awareness of emergency indicators, and the changes were sustained without additional resources.

Discussion

The quality improvement initiative demonstrated that simple workflow redesigns, staff engagement, and role clarity significantly improved referral efficiency and documentation. The intervention aligned with Universal Health Care priorities and national mandates for continuous quality improvement. Its success highlights the value of resident-led coordination and checklists in public hospital settings.

Conclusion

Structured, low-cost interventions markedly improved emergency referrals from an outpatient department. The approach may be replicated in other public hospitals and adapted to different intrahospital transfers. Sustained improvements require integration into routine clinic operations and continued monitoring.

## Introduction

Timely referral from outpatient clinics to the emergency department (ED) is essential in the early identification and management of life-threatening conditions. In both local and international practice, delays in referral can lead to poor outcomes for cases such as myocardial infarction, stroke, and severe respiratory distress, where every minute counts [1-3]. For example, each 30-minute delay in treating ST-segment elevation myocardial infarction (STEMI) is associated with a 7.5% relative increase in one-year mortality [1], and an untreated ischemic stroke can destroy an estimated 1.9 million neurons per minute [2]. Family physicians frequently encounter such emergencies in outpatient settings, including acute coronary syndrome and acute dyspnea [4,5]. Studies have documented that primary care clinics manage urgent cases on a regular basis. In one survey, general practitioners in rural Australia handled a median of eight emergency events per year, with nearly all practices seeing at least one emergency annually [5,6]. Pediatric emergencies in ambulatory practice, although less common, also demand prompt recognition and transfer, as even rare critical illnesses in children, such as sepsis or acute respiratory failure, require immediate intervention [4].

Outpatient providers must be ready to initiate emergency care and arrange rapid referrals when needed. At East Avenue Medical Center (a public tertiary hospital in the Philippines), an increasing number of patients seen in the Department of Family and Community Medicine’s outpatient clinic were being referred to the ED for further evaluation and management. A review of records from previous years (prior to 2023) showed a rising trend in such emergency referrals. Despite the growing volume, the turnaround time for completing the referral process remained prolonged, and compliance with standard documentation requirements was inconsistent. For instance, baseline audits revealed that while all referred patients had initial vital signs recorded, many charts lacked full documentation of the patient’s history or physical examination findings, and some referrals were not promptly endorsed to the ED staff.

These inefficiencies in the referral workflow prompted the department to undertake a retrospective before-and-after quality improvement (QI) project to identify problem areas and implement corrective measures. The aim of this study was to evaluate the existing referral system and implement structured interventions to improve the speed and quality of referrals from the outpatient clinic to the ED. Specifically, we targeted a reduction in referral turnaround time and an increase in compliance with the referral process checklist (covering documentation and communication steps). By optimizing the referral workflow and increasing staff awareness, the goal was to ensure faster triage, better interdepartmental communication, and improved patient safety for emergent and urgent cases.

This QI initiative was guided by the Plan-Do-Study-Act (PDSA) cycle, consistent with the Model for Improvement framework advocated for continuous quality improvement in Philippine primary care [7]. The project aligns with national efforts to strengthen quality of care under the Universal Health Care Act (Republic Act No. 11223), which emphasizes timely, effective, patient-centered services, and the Department of Health’s mandate for continuous quality improvement in all health facilities [8].

## Materials and methods

Study design and setting

This project was a retrospective quality improvement (QI) initiative conducted in the Department of Family and Community Medicine of East Avenue Medical Center, a tertiary government hospital located in Metro Manila. The methodology and findings were reported in accordance with the Standards for Quality Improvement Reporting Excellence (SQUIRE 2.0) guidelines [9]. The primary goal was to evaluate and improve the workflow for emergency referrals from the Family Medicine Outpatient Department to the Emergency Department (ED). The study period covered March 1 to June 30, 2023.

Patients and sampling

Patients included in the review were those identified in the outpatient clinic as having urgent or emergent conditions that warranted ED transfer. An “emergency referral” was defined as any patient seen in the Family Medicine clinic who was referred to the ED on the same visit due to conditions such as chest pain suggestive of myocardial infarction, stroke symptoms, severe difficulty breathing, hemodynamic instability, or other acute medical issues beyond the scope of outpatient management. Convenience sampling of available medical records was used. A total of 40 patient charts were reviewed: 20 consecutive emergency referrals prior to the intervention (baseline group, referred in March 2023) and 20 consecutive referrals after the intervention (post-intervention group, referred in late June 2023).

Ethical considerations

This project was carried out as part of an internal departmental quality improvement activity. All data were collected from existing medical records, with no direct patient intervention performed. No identifiable personal information was recorded or stored in any study materials. In line with institutional policy and national guidelines, internal reviews of clinical workflows using anonymized retrospective data are typically exempt from formal Institutional Review Board (IRB) review. Therefore, IRB approval was not required for this project. All activities were conducted in accordance with accepted ethical standards for healthcare quality improvement initiatives.

Definitions and outcome measures

Referral turnaround time was defined as the duration from the patient’s initial assessment in the outpatient clinic (marked by the recording of vital signs by clinic staff) to the formal acknowledgment of receipt by the ED triage nurse or officer. This interval included the time needed for medical evaluation, preparation of referral documentation, patient transport to the ED, and communication of handoff information.

Compliance with workflow documentation was assessed using a standardized referral process checklist developed by the department. This checklist included the following key components: (a) complete recording of vital signs in the clinic; (b) documentation of relevant medical history; (c) a focused physical examination; (d) preparation of a written referral form or letter with clinical details; and (e) confirmation of verbal endorsement to the ED triage staff. Each checklist item was marked as “yes” or “no” depending on completion. For each referral, the number of completed items was tallied to determine the compliance percentage. For example, a referral with four out of five completed steps scored 80% compliance. Average compliance scores were computed separately for pre- and post-intervention periods. Specific steps that were most frequently missed were also noted.

To provide clinical context, emergency severity at triage was categorized using the Canadian Triage and Acuity Scale (CTAS), a five-level system widely used in emergency departments to assess acuity levels: Level 1, resuscitation; Level 2, emergent; Level 3, urgent; Level 4, less urgent; and Level 5, non-urgent [10]. In this study, cases referred to the ED typically fell under CTAS Levels 1 to 3, which represent high-acuity categories. Although we did not assign individual CTAS scores to each case, these guidelines informed the types of clinical presentations deemed appropriate for immediate referral [10].

Use of referral tools

To ensure standardization and accurate time tracking, the department employed two main tools. The first was the Routing Slip for Emergency Department Referral, a standardized form used to document the timing of each step in the referral process. This included the recording of initial vital signs, the completion of medical history and physical examination, and the patient’s transfer and endorsement to the Emergency Department (ED). For each action, the start and end times were noted, and the form was signed by both the referring Family Medicine resident and the ED triage officer. This routing slip served as the primary reference for calculating referral turnaround time and confirming that the endorsement process had been properly completed, as summarized in Table 1.

**Table 1:** Routing Slip for Emergency Department Referral

Field	Value	
Name		
Age		
Sex		
Hospital number		
Workflow step	Time start	Time end
Taken vital signs and anthropometric measurements of the patient		
Completed medical history and physical examination		
Received referral letter and arrived at the Emergency Department accompanied by the nurse/nursing attended and resident-in-charge		
Endorsed by	Received by	
Family Medicine Resident-in-Charge	Emergency Department Triage Officer	

The second tool was the Emergency Department Referral Compliance Checklist, which was developed to ensure adherence to documentation standards and referral protocols. This checklist contained 13 required items covering key components of emergency referrals, including patient identification, documentation of vital signs, a complete medical and social history, findings from the physical examination, completion of the referral letter, and confirmation that the patient was properly handed off to the ED staff. For each item, the referring team marked whether it was “done,” “not done,” or “not applicable,” allowing the department to assign a compliance score for each case based on the number of completed items. The checklist was incorporated into the patient’s chart and collected after the intervention period for auditing purposes. A summary of the checklist components is shown in Table 2.

**Table 2:** Emergency Department Referral Compliance Checklist

Criteria	Done	Not done	N/A
1. Vital signs and anthropometrics taken			
2.1. Name			
2.2. Age			
2.3. Sex			
2.4. Address			
2.5. Contact number			
2.6. Hospital number			
2.7. Birthday			
2.8. Marital status			
2.9. Religion			
3. Chief complaint			
4. History of present illness			
5. Past medical history			
6. Family history			
7. Sexual history			
8. Obstetrics/gynecologic history			
9. Review of systems			
10. Physical examination			
11. Referral letter to Emergency Department			
12. Accompanied and endorsed patient to ER Triage Officer			
13. ER Triage Officer signed the receiving copy of referral letter and routing slip			

Intervention development

The intervention phase (April-May 2023) included several structured activities aimed at improving referral efficiency and documentation. We first convened focused group discussions with the Family Medicine residents and consulting staff to map out the existing referral process, identify bottlenecks, and solicit suggestions. Commonly cited issues included delays in locating nurses to accompany patients to the ED, incomplete charting due to the haste of transfers, and uncertainty among junior trainees about which cases required ED referral. We then implemented our interventions based on staff input (our “Do” phase of the PDSA cycle).

Our first step implemented was protocol training for all outpatient clinic staff (residents, interns, and nurses). This included reinforcing the recognition of red flag symptoms (e.g., chest pain with diaphoresis, hypotension, and neurological deficits) that should trigger immediate ED referral, following guidelines adapted from CTAS and local emergency care algorithms. We emphasized the importance of completing the referral checklist steps even in urgent situations through brief drills and simulations.

Our second step was triage coordination, where we introduced a system of assigning a senior Family Medicine resident to the outpatient triage window every clinic day. This resident’s role was to assist in the early identification of any walk-in patients who appeared critically ill, ensure vital signs are promptly taken, and facilitate rapid referral for those patients. The assigned resident would essentially act as a liaison, calling the ED triage officer ahead of time to alert them of an incoming referral whenever a high-acuity case was identified.

Our third step was personal endorsement. We instructed that for every referral, the resident (or intern/clerk under supervision) must personally accompany the patient to the ED and give a face-to-face endorsement to the ED triage nurse or physician. A phone call to the ED prior to transfer was also done for Level 1 or 2 cases (e.g., suspected myocardial infarction or stroke) to expedite readiness on the ED side. We provided an endorsed referral form to be signed by the ED receiving staff to document that the handoff was completed.

Our fourth step was making changes to nursing workflows. The outpatient department nursing staff were reoriented to prioritize assisting with emergency transfers. We established that a nurse from the clinic should be readily available to transport the referred patient (e.g., via wheelchair or stretcher) and that the nurse would carry the referral letter and relay vital information if needed. The nursing supervisors adjusted scheduling to ensure at least one float nurse in the vicinity of the Family Medicine clinic at all times for this purpose.

Lastly, we did a checklist integration into the standard outpatient consultation form for referred patients to prompt physicians to complete each item. The checklist had to be ticked off and attached to the chart before the patient was sent to the ED. The department clerk was tasked with collecting these checklists for audit.

These interventions were put into practice by May 2023. Throughout the implementation, we monitored informally for any issues (the “Study” phase). For example, we gathered feedback that the personal endorsement was working well on weekdays, but interns on duty during evenings initially forgot to call the ED in advance; we addressed this with additional reminders and by including this step in our sign-out protocols (“Act” phase adjustments).

Data collection and analysis

Data were manually extracted from patient charts, referral forms, and triage logs using a standardized abstraction form. For each case, we recorded the timestamp of initial vital signs in the clinic and the time of ED triage receipt (noted on ED log or referral form) to compute turnaround time in minutes. We also recorded the completion of each checklist item (yes/no). The data abstractors (residents not involved in the care of those patients) underwent a unified orientation to ensure consistent interpretation of documentation. Inter-rater reliability was addressed by double-checking a sample of charts by two independent reviewers, resolving any discrepancies by consensus. We summarized quantitative outcomes with descriptive statistics. Mean or median turnaround times were calculated for pre- and post-intervention groups (the distribution was approximately normal, so mean was used for simplicity). For compliance, we calculated the percentage of completed items overall and for each item. Given the small sample size, formal hypothesis testing was not emphasized; this was primarily a before-and-after audit. However, for illustrative purposes, we note percentage point improvements. The focus was on practical significance (effect size) in the clinical process.

Data extracted from patient records included vital signs, medical history components, and completeness of referral documentation, as summarized in Table 3. Turnaround times and compliance scores were computed using timestamps on the referral routing slip and the 13-item compliance checklist.

**Table 3:** Extracted Data Points for Each Emergency Referral Case HPI: history of present illness, PMH: past medical history, FHx: family history, OB-GYN: obstetrics/gynecology, ROS: review of systems, PE: physical examination, ED: emergency department, OPD: outpatient department

Variable	Description
Patient demographics	Age, sex, and hospital number
Reason for referral	Presenting complaint (e.g., chest pain, dyspnea, and stroke symptoms)
Vital signs taken	Time and completeness of vital signs documentation
Medical history components	Chief complaint, HPI, PMH, FHx, sexual/OB-GYN history, and ROS
Physical examination	Documentation of focused PE relevant to referral
Referral documentation	Presence and completeness of referral letter
Verbal endorsement	Documentation of personal or phone endorsement to the ED
ED acknowledgment	Confirmation of receipt via signed routing slip
Turnaround time (minutes)	Time from initial OPD contact to ED endorsement
Total checklist compliance (out of 13 items)	Count of completed items from the referral checklist

## Results

A total of 40 emergency referral cases were reviewed, with 20 cases during the pre-intervention (baseline) period and 20 cases during the post-intervention period. The most common reasons for referral were acute coronary syndrome (n=8), suspected stroke (n=6), hypertensive urgency or severe hypertension (n=5), and acute respiratory distress (n=5). The remaining cases involved other emergent conditions such as sepsis or trauma. Upon arrival at the emergency department (ED), most patients were triaged as high-acuity, with the majority classified under Canadian Triage and Acuity Scale (CTAS) Levels 2 or 3. A few were categorized as CTAS Level 1, requiring immediate critical care.

Turnaround time

Before the intervention, the average turnaround time from initial outpatient assessment to handoff at the ED was 43.05 minutes (range: 30-60 minutes). After the intervention, the average turnaround time decreased to 15.75 minutes (range: 10-25 minutes). This represented a 63% reduction in transfer time. Notably, 18 out of 20 post-intervention referrals (90%) were completed in under 20 minutes, compared to none in the pre-intervention group. This improvement was attributed to earlier case recognition by the triage resident and more efficient processing once a referral decision was made. These results are summarized in Table 4.

**Table 4:** Comparison of Key Metrics Before and After Intervention

Metric	Pre-intervention	Post-intervention
Average turnaround time (minutes)	43.05	15.75
Documentation compliance (%)	75.15	98
Complete medical history (number)	14	20
Complete physical examination (number)	14	20
Verbal endorsement (number)	10	19

Compliance with the referral process

Checklist compliance also improved. During the pre-intervention audit, the average compliance was 15.03 out of 20 (75.15%), indicating that about three-quarters of the required referral steps were completed. Specific deficiencies included incomplete documentation of medical history and physical examination (n=14, 70%), and lack of verbal endorsement to ED staff (n=10, 50%).

In the post-intervention period, overall compliance increased to 19.6 out of 20 (98%). All cases (n=20) had complete documentation of vital signs, medical history, and physical examination. Verbal endorsements were documented in 19 out of 20 cases (95%). In the remaining case, the referring resident missed the phone call, but the accompanying nurse completed the handoff. All cases included a formal referral letter or form. These metrics are also presented in Table 2.

**Figure 1:**
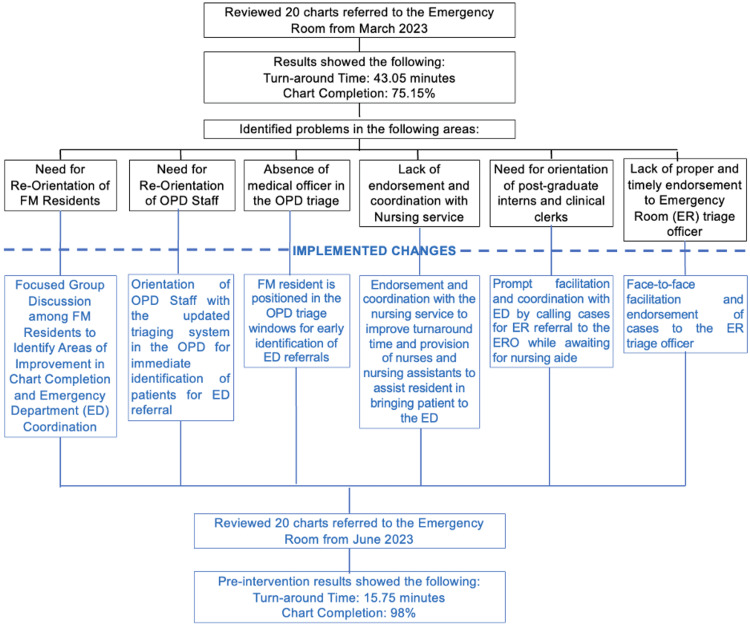
Process Workflow of the Interventions for Emergency Referral FM: Family Medicine, OPD: outpatient department, ER: emergency room, ED: emergency department

Figure 1 shows the specific system changes made to close these compliance gaps. It links observed problems, such as incomplete charting, absence of a medical officer in triage, and delays in nurse coordination, with specific interventions implemented. These included staff reorientation, resident triage assignment, and direct face-to-face endorsement to the ED. These changes led to reduced average turnaround time and improved checklist compliance.

Documentation and communication improvements

The quality of referral documentation improved. Before the intervention, several referral letters were either missing or incomplete. After the intervention, referral forms were standardized and consistently included structured summaries of clinical findings and initial outpatient management. Face-to-face endorsements and advance calls to the ED allowed receiving teams to prepare appropriately.

Staff familiarity with referral indicators improved as well. During the post-intervention period, interns and clerks were able to identify and refer emergency cases independently. This behavior was not commonly observed during the baseline period and reflected improved orientation and understanding of triage protocols.

Adverse events and feasibility

No adverse events or unintended consequences were reported. None of the post-intervention cases experienced clinical deterioration due to referral delays. In contrast, one pre-intervention patient with unstable angina had a delay of nearly 60 minutes before transfer to the ED.

The intervention did not require new funding or additional staffing. All improvements were achieved through clearer protocols and reallocation of existing personnel. The changes were well-accepted by both the outpatient and ED teams, supporting feasibility and sustainability.

## Discussion

The results of this quality improvement initiative showed that a structured, team-based intervention significantly improved the efficiency and reliability of emergency referrals from an outpatient clinic to the emergency department. We achieved a meaningful reduction in referral turnaround time, from 43 minutes to 15 minutes on average. This was critical in a busy tertiary government hospital where patient volume was high and timely management of emergent conditions could be lifesaving. In acute care, even small time improvements could affect outcomes in time-sensitive conditions. For cases like myocardial infarction and stroke, expedited transfers facilitated earlier definitive treatment, in line with the principle that “time is muscle” or “time is brain” [1,2].

The increase in referral process compliance from 75% to 98% reflected improved standardization and accountability. By introducing a simple checklist and promoting thoroughness even in urgent cases, we addressed documentation gaps that previously led to information loss. In our baseline audit, incomplete histories and a lack of direct communication often left ED staff uncertain about incoming patients. After the intervention, nearly all referrals were accompanied by complete written and verbal handoffs, which prior literature identifies as essential for safe transitions of care [11].

Similar improvements in documentation and communication have been reported in other low-resource settings after implementing structured referral checklists [12,13]. Our approach relied on low-cost and low-technology solutions, primarily human factors interventions such as staff training, role designation, and checklist use. This strategy was especially relevant in a setting where hospitals often lack advanced IT systems or additional personnel [14]. Our experience showed that empowering existing staff with clear protocols and responsibilities led to substantial gains, consistent with findings that frontline staff, particularly nurses, play a critical role in improving hospital quality and efficiency when actively engaged in process improvement [15].

This approach was consistent with other quality improvement projects in low- and middle-income countries. For example, a recent project in a South Indian hospital used Plan-Do-Study-Act cycles and staff feedback to reduce waiting times and improve ED workflows without additional funding or equipment [12]. Like our intervention, it focused on redesigning processes and training staff, resulting in measurable improvements in emergency care. However, compared to those studies, our project addressed a different referral point (internal transfers from outpatient to emergency settings), where published literature, especially in the Philippine context, remains sparse.

To our knowledge, no peer-reviewed studies in the Philippines have described internal quality improvement initiatives targeting the outpatient-to-ED referral process. Most existing efforts focus on vertical referrals between facilities (e.g., stroke fast lanes or STEMI pathways) or specific disease programs. This lack of published models for internal emergency coordination within Philippine public hospitals makes this study a novel contribution. It provides a practical and scalable microlevel system improvement aligned with national goals for quality under the Universal Health Care Act.

Our project’s use of a resident-led triage coordination role also proved scalable. Residency programs in family and emergency medicine could integrate similar roles into their training structure. In our case, residents assumed responsibility for triage, which improved patient care and enhanced training in emergency recognition and systems-based practice. This adds an educational component that strengthens residency competencies in quality and safety, aligning with the shift toward system-based approaches to patient safety [16].

We also observed stronger collaboration between departments. Initially, there was a gap between the outpatient and ED teams that contributed to communication delays. The project helped foster a more collaborative environment, with outpatient staff anticipating ED needs and ED staff recognizing the improved workflows. This interdepartmental cooperation was essential. As noted by Al Qassim et al. in a review of ED referrals from primary care, maintaining quality during transfers requires coordination and shared expectations between providers [11]. Our intervention supported these shared expectations through direct communication and feedback.

Compared to other published QI efforts, our project was short in duration and limited to a single department. Still, it addressed a specific gap in the Philippine setting, where published work on internal ED referral systems in public hospitals is limited. Previous efforts have focused more on referral systems between facilities or for specific conditions such as STEMI and stroke [17].

This study offered a microlevel model that improved internal processes at the point of care, recognizing that even small system changes can have ripple effects in the complex, dynamic nature of healthcare delivery [18]. It supported the Department of Health’s Continuous Quality Improvement mandate under the Universal Health Care Act, which requires facilities to regularly assess and improve service delivery [17]. The workflow we implemented can serve as a low-cost model for internal coordination that is replicable in other resource-constrained public hospitals.

By improving a common but critical process like emergency referrals and showing measurable results, our project offered a model that other departments or institutions could adapt. The same structure could be used in referrals from community health centers to hospitals, or from general wards to intensive care units, to ensure clear communication and timely transfers. Core principles such as early emergency recognition, structured documentation, verbal handoff, and role clarity apply across multiple settings and levels of care.

A major strength of our project was its inclusive approach. Physicians, nurses, and clerks helped design and carry out the changes. This likely contributed to better adoption of the new process. We also had strong administrative support once the results became clear. The hospital is now considering formalizing the triage officer role in outpatient staffing, which supports long-term sustainability. For a QI initiative to continue, it often needs to be embedded in hospital policy. This may include official memos, staffing adjustments, or regular monitoring.

We recommended that the referral checklist become a standard part of clinic records for any emergent case, with periodic audits to monitor compliance. We also suggested that the ED provide short feedback to the referring team on referral quality and outcomes. This would help improve triage decisions and promote a feedback culture.

This project had limitations. First, the sample size was small due to the short time frame and number of cases. We did not conduct formal hypothesis testing. While our results were large in magnitude, they should be interpreted as observational rather than definitive estimates. Second, this was a single-center project. Generalizability to other settings, such as private clinics or rural health centers, may be limited. However, the principles of early emergency recognition, clear referral steps, and direct communication are widely applicable and aligned with international best practices in emergency care [19]. Third, we did not assess clinical outcomes such as changes in morbidity or mortality before and after the intervention. Larger studies would be needed to evaluate those effects. Still, there is strong evidence that faster treatment leads to better outcomes in emergencies such as myocardial infarction, stroke, and sepsis [1,3].

Future directions could include linking this workflow to clinical outcomes, such as door-to-needle time for stroke thrombolysis. Despite its limitations, our experience clearly showed that focused improvements in process can make acute care more efficient and safer. In low-resource environments, QI projects like this are often more impactful than costly technology. They also empower frontline staff to take ownership of improvements. This project started when residents identified a problem and followed through with minimal resources. It offers a model that can inspire other teams to do the same.

## Conclusions

In summary, implementing structured improvements in the referral process from the Family Medicine outpatient clinic to the ED led to markedly faster transfer times and more consistent adherence to referral protocols. By the end of the intervention, nearly all emergency referrals were accomplished with complete documentation and within a quarter of the time previously required. These improvements enhance patient safety and care continuity during critical referrals. To maintain improvements, we recommend that the hospital formalize the changes: for example, maintain a designated resident or staff member for triage coordination during clinic hours, continue regular training and orientation of new staff on emergency referral procedures, and keep using the checklist as a mandatory part of referral documentation. Moreover, integrating this referral workflow into hospital policy (and the orientation of rotating interns and clerks) will help ensure it becomes standard practice.

This quality improvement project demonstrates that even in a resource-limited setting, a committed team using QI methodology can significantly improve an important aspect of patient care. Other departments and health facilities might adapt this model to improve their own referral systems or similar interdepartmental processes. Ultimately, small system changes, such as streamlining referrals, contribute to the larger goal of a safer, more efficient health system under Universal Health Care. Continued monitoring and iterative improvement will be key, but the successes achieved provide a strong incentive to uphold these practices. We believe that assigning clear responsibility for ED referrals and fostering a culture of timely communication will remain crucial as patient volumes grow and as we strive to deliver responsive emergency care starting from the frontlines of primary care.
